# A multi-centre randomised Phase III trial of radioembolisation (RE) combined with oxmdg compared with oxmdg alone as first-line therapy for unresectable liver-only or liver-dominant metastatic colorectal cancer (CRC)

**DOI:** 10.1186/1745-6215-14-S1-P21

**Published:** 2013-11-29

**Authors:** Nicola Maycock, Susan Dutton, Ricky Sharma

**Affiliations:** 1 OCTRU, Oxford, UK; 2 OCTO, Oxford, UK; 3 CSM, Oxford, UK; 4 Department of Oncology, Oxford, UK; 5 University of Oxford, Oxford, UK

## Background

Colorectal cancer (CRC) is the third most common malignancy worldwide; the leading cause of death from this disease is progression of liver metastases. Radio-embolisation (RE) with yttrium-90 microspheres is a local radiotherapy treatment targeting liver metastases. The FOXFIRE trial has randomised over 200 participants in the UK and will be open to recruitment until May 2014.

## Methodology

Eligible patients have non-resectable liver metastases from histologically confirmed CRC with no or limited extra-hepatic disease and no prior treatment for metastatic disease. Each patient is randomised 1:1 to standard chemotherapy (Oxaliplatin, Folinic Acid, 5-Flurouracil) or chemotherapy plus RE, using minimisation with stratification factors: centre, liver involvement, presence of extra-hepatic disease and intention to treat with a biological agent.

## Recruitment issues

• Slow site activation: this trial involves complex radiological intervention, involving multi-disciplinary team working. These teams had to be formed for the trial. This caused complex negotiations facilitated by the trials office to encourage communication between different departments to ensure adequate quality of care.

• Change to current the treatment paradigm affecting recruitment: a protocol amendment was necessary to allow the use of biological agents (cetuximab or bevacizumab) with intent-to-treat with a biological agent added as a stratum to the randomisation.

## Conclusion

The key to improving recruitment has been regular communication with the site staff, newsletters, adapting to changing treatment paradigms, opening more sites than originally planned and providing publicity via the local research networks.

## Funding

Bobby Moore Fund of Cancer Research UK and Sirtex Medical Ltd.

